# Using Genetic Programming with Prior Formula Knowledge to Solve Symbolic Regression Problem

**DOI:** 10.1155/2016/1021378

**Published:** 2015-12-24

**Authors:** Qiang Lu, Jun Ren, Zhiguang Wang

**Affiliations:** Department of Computer Science and Technology, China University of Petroleum, Beijing 102249, China

## Abstract

A researcher can infer mathematical expressions of functions quickly by using his professional knowledge (called Prior Knowledge). But the results he finds may be biased and restricted to his research field due to limitation of his knowledge. In contrast, Genetic Programming method can discover fitted mathematical expressions from the huge search space through running evolutionary algorithms. And its results can be generalized to accommodate different fields of knowledge. However, since *GP* has to search a huge space, its speed of finding the results is rather slow. Therefore, in this paper, a framework of connection between Prior Formula Knowledge and *GP* (*PFK-GP*) is proposed to reduce the space of *GP* searching. The PFK is built based on the Deep Belief Network (*DBN*) which can identify candidate formulas that are consistent with the features of experimental data. By using these candidate formulas as the seed of a randomly generated population, *PFK-GP* finds the right formulas quickly by exploring the search space of data features. We have compared *PFK-GP* with Pareto *GP* on regression of eight benchmark problems. The experimental results confirm that the *PFK-GP* can reduce the search space and obtain the significant improvement in the quality of SR.

## 1. Introduction

Symbolic regression (SR) is used to discover mathematical expressions of functions that can fit the given data based on the rules of accuracy, simplicity, and generalization. As distinct from linear or nonlinear regression that efficiently optimizes the parameters in the prespecified model, SR tries to seek appropriate models and their parameters simultaneously for a purpose of getting better insights into the dataset. Without any prior knowledge of physics, kinematics, and geometry, some natural laws described by mathematical expressions, such as Hamiltonians, Lagrangians, and other laws of geometric and momentum conservation, can be distilled from experimental data by the Genetic Programming (*GP*) method on SR [1].

Since SR is an NP-hard problem, some evolutionary algorithms were proposed to find approximate solutions to the problem, such as Genetic Programming (*GP*) [2], Gene Expression Programming (GEP) [3], Grammatical Evolution (GE) [4, 5], Analytic Programming (AP) [6], and Fast Evolutionary Programming (FEP) [7]. Moreover, recent researches in SR problem have taken into account machine learning (ML) algorithms [8–10]. All of the above algorithms randomly generate candidate population. But none of them can use various features of known functions to construct mathematical expressions adapted for describing the features of given data. Therefore, these algorithms may exploit huge search space that consists of all possible combinations of functions and its parameters.

Nevertheless, a researcher always analyzes data, infers mathematical expressions, and obtains results according to his professional knowledge. After getting experimental data, he observes the data distribution and their features and analyzes them with his knowledge. Then, he tries to create some mathematical models based on natural laws. He can obtain the values of coefficients in these models through regression analysis methods or other mathematical methods. And he evaluates the formulas which are mathematical models with the values by using various fitness functions. If the researcher finds some of the formulas that fit the experimental data, he can transform and simplify these formulas and then obtain the final formula that can represent the data. Furthermore, his rich experience and knowledge can help him to reduce the searching space complexity so that he can find the best fit mathematical expression rapidly. As the researchers use their knowledge to discover the best fitted formulas, the methods that inject domain knowledge into the process of SR problem solving have been proposed to improve performance and scalability in complex problem [11–13]. The domain knowledge, which is manually created by the researcher's intuition and experience, is of various formulas which are prior solutions to special problems. If the domain knowledge automatically generates some fitted formulas that are used in evolutionary search without the researcher involvement, the speed of solving SR problem will be quickened. A key challenge is how to build and utilize the domain knowledge just like the researcher does.

**Figure 1:**
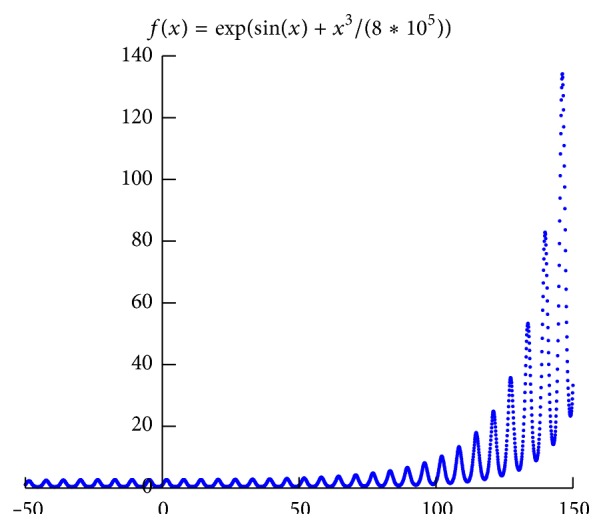
The function *f*(*x*) = exp⁡(sin⁡(*x*) + *x* ^3^/(8*∗*10^5^)).

In this paper, we present a framework of connection between Prior Formula Knowledge and* GP* (*PFK-GP*) to address the challenge:

(i) We classify a researcher's domain knowledge into PFK Base (*PFKB*) and inference ability after analyzing the process that a research discovered formulas from experimental data (Section 2).* PFKB* contains two primary functions: classification and recognition. The aim of two functions is to generate feature functions which can represent the feature of experimental data.
(ii) In order to implement classification, we use the deep learning method* DBN* [14, 15] which, compared with other shallow learning methods (Section 3.1), can classify experimental data into a special mathematical model that is consistent with data features. However, the classification method may lead to overfitting because the method can only categorize experimental data into known formula models which come from the set of training formula models.
(iii) Therefore, recognition is used to overcome the overfitting. It can extract mathematical models of functions that can show full or partial features of experimental data. Three algorithms* GenerateFs*,* CountSamePartF,* and* CountSpecU* (see Algorithms 2, 3, and 4) are designed to implement recognition. For example, from the dataset generated by *f*(*x*) = exp⁡(sin⁡(*x*) + *x* ^3^/(8*∗*10^5^)), the basic functions sin, exp, and cube can be found by the above three algorithms. In Figure 1, the function sin shows the periodicity of data, and exp or cube shows the growth rate of data. Therefore, these basic functions (called feature functions) can describe some features of the dataset.
(iv) The inference ability is concluded to the searching ability of evolutionary algorithm. As researches infer mathematical models,* GP* is used to combine, transform, and verify these models. These feature functions that are generated by* PFKB* are selected to be combined into the candidate population in the light of algorithm* randomGenP* (see Algorithm 5). With the candidate population,* GP* can get convergent result quickly because it searches answers in a limit space which is composed of various feature functions. Through experiment on eight benchmark problems (Table 5 *E*_1_–*E*_8_), the results demonstrate that* PFK-GP*, compared with Pareto optimization* GP* [16, 17], shows the significant improvement in accuracy and convergence.

## 2. Background

### 2.1. Definition and Representation of Mathematical Expression

In this section, we will define concepts about SR problem and show how to represent these concepts by applying* BNF* expression. For SR problem, the word “formula” is the general term which describes mathematical expression that fits the given data. We define a formula model is a special mathematical model in which formulas have the same relationships and variables except for different coefficient values. Relationships can be described by operators, such as algebraic operators, functions, and differential operators (http://en.wikipedia.org/wiki/Mathematical_model). Therefore, a formula model is a set where each element is a formula. For example, the two formulas 0.1*∗*sin⁡(*x*) + 0.7*∗*log⁡(*x*) and 0.3*∗*sin⁡(*x*) + 0.9*∗*log⁡(*x*) belong to the formula model *a*_1_ *∗*sin⁡(*x*) + *a*_2_ *∗*log⁡(*x*). Data that are represented by different formulas in one formula model may have similar features which are data distributions, data relationships between different variables, data change laws, and so on, because these formulas have the same relationships.

In order to represent a formula model and its corresponding formulas, we define the following* BNF* expressions:

 
*F*≔*C*∣*S*,
 
*C*≔*S*"("*C*")"∣*S*,
 
*S*≔*B”*("*AX*, *AX”*)"∣*U”*("*AX”*)",
 
*B*≔"+"∣"−"∣"*∗*"∣"/",
 
*U*≔*”*sqrt*”*∣*”*log*”*∣*”*tanh*”*∣*”*sin*”*∣*”*cos*”*∣*”*exp*”*∣*”*tan*”*∣*”*abs*”*∣*”*quart*”* ∣ *”*cube*”*∣*”*square*”*⋯,
 
*A*≔*a*_1_∣*a*_2_∣*a*_3_∣ ⋯ ∣*a*_*n*_,
 
*X*≔*x*_1_∣*x*_2_∣*x*_3_∣ ⋯ ∣*x*_*m*_,

where *F* is a formula model. *X* is a parameter set. *A* indicates a coefficient set. *B* is a set of binary functions, while *U* is a set of unary functions. *S* is a set of atomic functions which does not contain any subfunctions. *C* is a set of complex functions which contains complex functions in *C* and atomic functions in *S*. With the above definitions, any formulas and its corresponding model can be shown by these* BNF* expressions. For instance, the formula exp⁡(sin⁡(*x*) + *x* ^3^/(8*∗*10^5^)) is represented by *F* and *C*, and its subfunction sin⁡(*x*) is represented by *U*. The constants 8, 3, and 10^5^ are shown by elements in *A*. With these *BNF* expressions, a formula model can be transformed into one tree. And the tree is a candidate individual in population of* GP* solving SR problem. Every subtree in the tree is a subformula which can show special data features. A subtree that shows features of experimental data is called feature-subtree. If a tree has more feature-subtrees, the tree is more likely to fit the data. How to construct the tree consisting of feature-subtrees is a key step in our method which is implemented by the algorithm* randomGenP* (see Algorithm 5).

### 2.2. The Process of Researcher Analyzing Data

**Figure 2:**
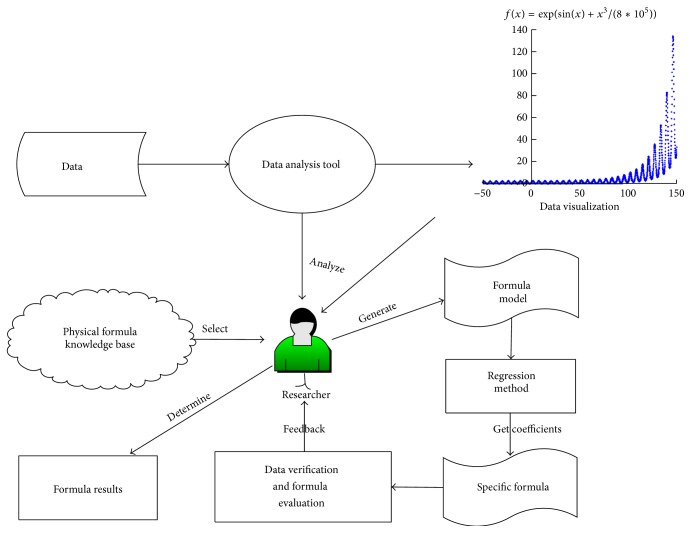
The process that researchers study the SR problem.

The process that a researcher tries to solve SR problems is shown in Figure 2. He depends heavily on his experience which is obtained through a long-term accumulation of study and research. After a researcher collected experimental data, he discovers regular patterns from data by using the methods of data analysis and visualization. He then constructs formula models which were consistent with these regular patterns according to his experiences. After that, he computes the coefficient values in formula models by using appropriate regression methods and obtains some formulas from different formula models. According to results of evaluating these formulas, he chooses the formula that is most fitted to the data. If the formula cannot represent data features, he needs to reselect a new formula model and do the above steps until one fitting formula is found.

We think the researcher's experience and knowledge have two roles in processing SR problem. One role is Prior Formula Knowledge (PFK) which can help a researcher to quickly find fitted formulas that match experimental data features. Through study and work, the researcher accumulates his domain knowledge of various characteristics of formula model. When the researcher observes experimental data, he can apply his domain knowledge to recognize and classify the data. The other is the ability of inference and deduction which can help the researcher to combine, transform, and verify mathematical expression. We conclude that the PFK contains two primary functions: classification and recognition.

**Figure 3:**
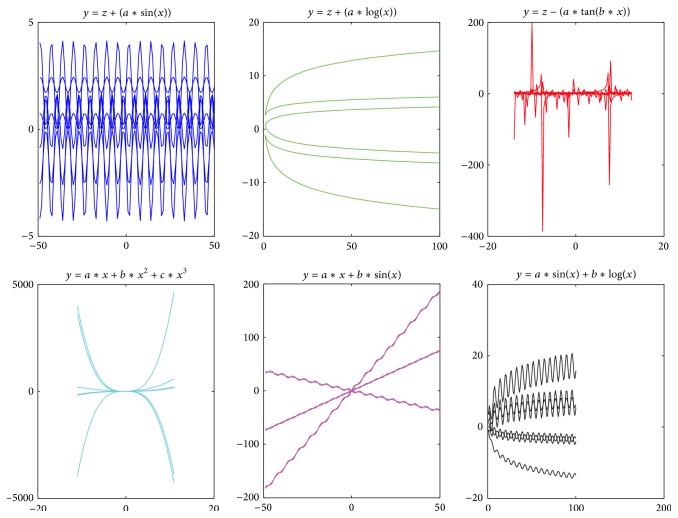
Curves are generated by six formula models with different coefficient value.

*Classification.* when experimental data features are in accord with characteristics of one formula model in PFK, the dataset can be categorized into the model. The prerequisite of classification is that different formula models have different characteristics in PFK Base. As shown in Figure 3, six families of curves are generated by six formula models taking different coefficient values. The curves in the same family show similar data features while the curves in different families show different data features. Therefore, we can infer that the curves (including surfaces and hypersurfaces) generated by different formula models can be classified according to their data features.

Although many machine learning algorithms such as linear regression [18], SVM [19], Boosting [20], and PCVMs [21] can be used to identify and classify data, it is difficult for these algorithms to classify these curves. That is because these algorithms depend on features that are extracted manually from data, while these features from different complex curves are difficult to be represented by a feature vector which is built based on the researcher's experiences. In contrast to these algorithms, DL can automatically extract features and have a good performance for the recognition of complex curves, such as image [15], speech [22], and natural language [23]. The* GenerateFs* algorithm (see Algorithm 2) based on* DBN* is shown to classify the data.

*Recognition.* Some formulas can represent remark features of curves generated by formula model. For example, after observing the curve in Figure 1, a researcher can easily infer that the formula sin or cos is one of formulas that constitute the curve because data in curve show periodicity. Therefore, these formulas are called feature functions that can be recognized or extracted by PFK. Algorithms* CountSamePartF* and* CountSpecU* (see Algorithms 3 and 4) are built to recognize the feature functions.

Recognition can help the researcher overcome overfitting of results that are generated by classification because classification can help researcher to only identify formula models from training set while recognition can help the researcher identify subformula models that are consistent with local data features.

The ability of inference and deduction is one of main measurements for evaluating performance of artificial intelligence methods. In the SR problem,* GP*, compared with other methods such as logical reasoning, statistical learning, and genetic algorithm, is a revolutionary method of searching fitting formulas because it can seek the appropriate formula models and their coefficient values simultaneously by evolving the population of formula individuals. Therefore, in the paper, we use* GP* as the method of inferring and deducing formulas.

To optimize* GP*, researchers have proposed various approaches, such as optimal parsimony pressure [24]; Pareto front optimization [17] and its age-fitness method [25] are used to control bloat and premature convergence in* GP*. In order to reduce the space complexity of searching formulas, the methods of arithmetic [26] and machine learning are injected into* GP*. In the paper, with the algorithm* randomGenP* (see Algorithm 5) about generating population and the method of Pareto front optimization,* PFK-GP* can research the formula model in the appropriate space and can find right formulas quickly.

## 3. Genetic Programming with Prior Formula Knowledge Base

### 3.1. Formula Prior Knowledge Base

**Figure 4:**
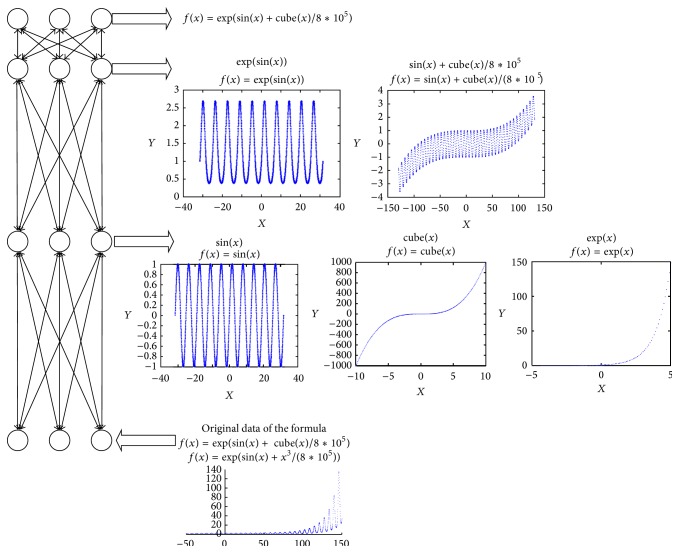
Illustration of the* DBN* framework.

**Figure 5:**
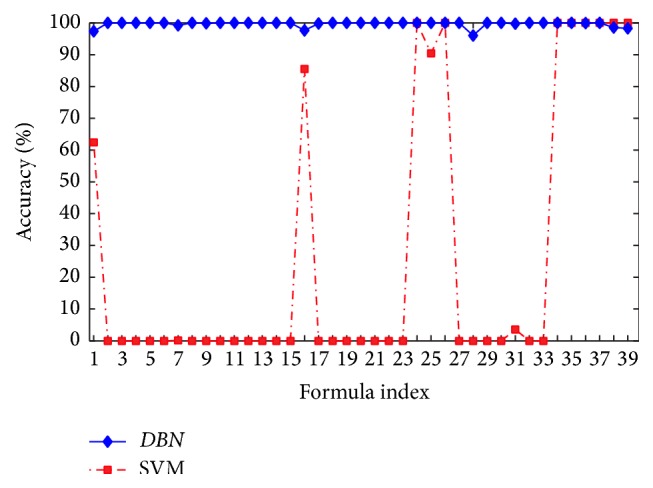
Accuracy result of SVM and* DBN* classifying *P*_1_–*P*_39_.

The FPK needs to have the ability of identifying and classifying the formula model *F* based on data features. Although the features between formula models are different, it is difficult to extract features from data which are generated by these models because different formula models represent seemly similar but different features. Based on the above definitions in formula model, the features among functions in set *S* are different. The features between the function *s* ∈ *S* and the function *c* ∈ *C* may be similar if *c* is the parameter of *s*. As shown in Figure 4, these functions sin⁡(*x*), cube(*x*), and exp⁡(*x*) belonging to *S* constitute *C*, such as exp⁡(sin⁡(*x*)) and sin⁡(*x*) + cube(*x*)/(8*∗*10^5^), and are shaped into the final function exp⁡(sin⁡(*x*) + cube(*x*)/(8*∗*10^5^)). sin⁡(*x*) shows periodicity in results; log⁡(*x*) shows slow variation trend in results; cube(*x*) shows high variation trend in results. So, these features in the three functions are different. However, there are similar features between cube(*x*) and sin⁡(*x*) + cube(*x*)/(8*∗*10^5^), because the two functions have high variation trend in result. The shallow learning method such as SVM can hardly identify complex features of functions shown in Figure 5.

In this paper,* DBN* is used to classify data into a special formula model according to features that are automatically extracted from data. Generally,* DBN* is made up of multiple layers that are represented by Restricted Boltzmann Machine (RBM). As RBM is an universal approximate of discrete models [27], we can speculate that* DBN* which has many layers of RBM can recognize the features of data generated by complex function just like Convolutional Neural Network (CNN) classifying image [28]. The process of* DBN* recognizing formulas is illustrated in Figure 4.* DBN* can extract features from data, layer by layer. So, the lower RBM layer can represent the simple functions and the higher RBM layer can transform the simple functions into more complex functions.

We use the data generated by the different formula models *F* (see Table 7) as training samples.* DBN* is trained by these samples. The model that is finally gained by* DBN* training methods is* PFKB*, which is aimed at identifying the formula model that can represent features of the data. The process of* DBN* training is outlined in algorithm* TrainDBN *(Algorithm 1) which uses the same steps as mentioned in [14, 15].

*PFKB* is only changed with formula models. If there are no new trained formula models in an application, the algorithm* TrainDBN* will not be executed. When the number of trained formula models is large enough, little new formula model will appear, and* PFKB* will seldom be changed. In the paper,* TrainDBN* is performed exactly once in order to generate* PFKB*.

### 3.2. Classification and Recognition with* PFKB*

In order to deal with the problem of how to classify and recognize formula model from data, we should consider the problem from two aspects. One situation is that data can be represented by a special formula model from* PFKB*, while the other one is that data cannot be represented by a formula model from* PFKB*. In the first case, we exploit* PFKB* to identify formula models of data by* DBN* classification. Based on ordered results of* DBN* classification, we gain a set of formula models (*Fs* = *f*_1_,…, *f*_*s*_) which are most similar to features of the data. The process that deals with the first case is outlined in algorithm* GenerateFs*. The algorithm is fast because* PFKB* has been built by* TrainDBN*, and *s* is small integer value.

In the second case, when a researcher observes laws that are hidden in experimental data, he often tries to find some formulas *C* which are consistent with partial features of the data. Therefore, we propose the two assumptions as follows.

**Assumption 1 .**

More formula models *f*s have the same subformula model* pf* in the set* Fs* which is the result of* GenerateFs* running, more strongly that the* pf* can express features of data.

In order to compute the same* pf* in* Fs*, we express the formula model as the string of expression and seek the same part of them by using intersection between the two strings (without considering the elements in sets* X* and* A*). Define the intersection between two expressions as follows:

(1) fi∩fj=c1,…,ck,cm∩cn=∅,m≠n,  cn∈C,  cn∉S,  1≤m,  n≤k,  1≤i,  j≤k.

For example, *f*_1_ = *z* + *a∗*cos⁡(*x*) + tan⁡(*x*)/(exp⁡(*x*) + log⁡(*x*)), *f*_2_ = *z* + *a∗*cos⁡(*x*) + abs(*x*)/(exp⁡(*x*) + log⁡(*x*)), *f*_1_∩*f*_2_ = {*z* + *a∗*cos⁡(*x*), exp⁡(*x*) + log⁡(*x*)}. The method, which obtains* pf* whose frequency of occurrence in* f* is larger than threshold* t*, is described as the algorithm* CountSamePartF*.

For verifying Assumption 1, we apply the dataset from *E*_0_ (see Table 5) as the testing set and get the identifying results *Fs* = {*P*_18_, *P*_15_, *P*_34_, *P*_11_, *P*_3_} from Table 7 through the algorithm* GenerateFs*. The intersections between the top two formulas *P*_18_∩*P*_15_ are (sqrt(*x*_0_))/(tan⁡(*x*_1_)) and (tan⁡(*x*_0_))/(exp⁡(*x*_1_)), which are partial mathematical expressions in *E*_0_. And we use the dataset from Table 6 to test *E*_0_ and get the identifying results *Fs* = {*T*_7_, *T*_6_, *T*_8_, *T*_9_, *T*_1_} through* GenerateFs* algorithm. The intersection between two expressions is as follows: *T*_7_∩*T*_6_ = {sqrt(*x*_0_)/tan⁡(*x*_1_)}, *T*_8_∩*T*_9_ = {tan⁡(*x*_0_)/exp⁡(*x*_1_), cos⁡(*x*_0_)*∗*sin⁡(*x*_1_)}. We find that the elements which have more frequency of occurrence in the intersections set are more likely to express some data features. The above two experiments illustrate that Assumption 1 is rational.

**Assumption 2 .**

If function *u* ∈ *U* exists in* Fs* obtained by* GenerateFs* and the number of the same *u* is larger than threshold *t*, we can conclude that *u* can show some local data features.

The function *b* ∈ *B* except *x* ^*y*^ is common function, which has a high probability of occurrence in mathematical expressions. Therefore, it is difficult to express special data features. Compared with *B*, the function *u* ∈ *U* can show obvious features of data. For instance, sin⁡(*x*) presents the periodicity of data and log⁡(*x*) represents data features about extreme increase or decrease. The method, which obtains the special function *u* that can show the local data features, is outlined as the algorithm* CountSpecU*.

For verifying Assumption 2, we also choose the dataset which are generated from *E*_0_ (see Table 5) as the testing data and apply the* CountSpecU* algorithm to calculate the special *u* among *Fs* = {*P*_18_, *P*_15_, *P*_34_, *P*_11_, *P*_3_}. The result of the algorithm is shown in Table 1. We find the result *specU* = {tan, cos, sqrt, exp, sin} (sin and cos are the operators of the same kind) is part of *E*_0_. Hence, we can discover that the* u* set, which is gained by the algorithm* CountSpecU*, can show local features of the dataset.

### 3.3. *GP* with Prior Formula Knowledge Base

In order to deal with SR problem,* GP* is executed to automatically composite and evolve mathematical expression. The process of* GP* is similar to the process that a researcher transforms formula models and obtains fitting formulas based on his knowledge. Since those algorithms in* PFKB*, which is created based on analyzing the process of how a research infers fitted formulas, can recognize formula models that are consistent with data features, we combine these formula models of* PFKB* recognizing into the process of* GP* in order to reduce the searching space and increase the speed of discovering right solutions.

When initializing* GP* algorithm, we can select candidate formulas from* Fs*,* C,* and* specU* as individuals in population of* GP*. The sets* Fs*,* C,* and* specU* are gained by the above algorithms in* PFKB*. Therefore, the* PFKB* is injected into the process of population generating. And this population can contribute to reserving data features as much as possible and reducing the searching space because these individuals commonly have good fitness value. With the population,* GP* algorithm can speed up the convergence and improve the accuracy of SR results. However, it may lead to the bias results. To overcome the problem, some random individuals must be imported into the population. The process of population creating is as follows.

Firstly, the elements in sets* Fs* and *C* are inserted into the population. Then, the set* specU* and the candidate function sets *B* and* U* are merged into the new candidate function queue* Q*. And the number of elements in* specU* is twice as much as the other elements in* Q* because *B* ∪ *C*⊆*specU*. Those elements in* specU* are more likely to be part of individuals in the population after applying the method* traditionalRandomIndividual* [16] which is designed to generate randomly *k* individuals from the special function set. At last, the rest of individuals of population are created by* traditionalRandomIndividual* with sets* B* and* U*. The process of population generating is described as the algorithm* randomGenP*.

Generally, |*Fs* | +|*C* | +|*specU* | <*n*/2, where *n* is the number of individuals in population. Furthermore, in order to enhance the affection of* PFKB* in the process of* GP* evolution, the method* randomGenP* is used to create new individuals in every few generations of evolutionary computation. Meanwhile, the method of Pareto front [17] is introduced into the algorithm* PFK-GP* to balance the accuracy against the complexity of model. The detail of algorithm* PFK-GP* is shown in Algorithm 6.

## 4. Experiments

In the experiments, we employ* DBN* in the DeepLearnToolbox [30] to classify formula models and build the algorithm* PFK-GP* based on GPTIPS [29]. The 39 formula models in Table 7 are composed of formulas from [31, 32] and some formulas are created by ourselves. The data generated by these 39 formula models is used as training data of algorithm* DBN* to create* PFKB*. The formula models in Table 5 are used to generate the testing data for verifying accuracy of algorithms* GenerateFs* and* PFK-GP*. The formula models in Table 6 are devoted to validating the two algorithms* CountSamePartF* and* CountSpecU* (see Algorithms 3 and 4).

For most formula models from Tables 5, 6, and 7, we sampled them by equal step taking their parameter values from the range [−49, 50]. For some particular formulas, we also sample them with a special equal step from special numerical scope. For example, the value *x* in sqrt(*x*) is in the range [0, 99], the value *x* in log⁡(*x*) ranges between 1 and 100. We create 500 groups of different parameters value in each formula model. The coefficients in these formula models are fetched with equal step from the range [−2.996  3.0]. When all coefficients of a formula model take special values, the formula model generates a formula, namely, a sample of the formula model. We create 7500 groups of different coefficients in each formula model. So, each formula model has 7500 samples where each sample has 500 groups of different parameters value. We take 6000 samples of these samples as training data and the others as test data.

We adopt* DBN* as the classification model and compare it with SVM that is implemented by the tool libsvm [33]. The training and testing data for the two algorithms are originated from formula models *P*_1_–*P*_39_. The parameter values in* DBN* and SVM are illustrated as Table 2. We take the first five formulas from* Fs* generated by* GenerateFs* as a result set of recognition. If the test formula is included in the set, we think that the recognition result is correct. The accuracy of recognition results of* DBN* and SVM is showed as Figure 5. The* DBN* method can help to classify all kinds of test data into its fitted formula models. However, the SVM method can only correctly classify several kinds of test data. The overall average accuracy of* DBN* classification is 99.65%, while the accuracy of SVM is 26.72%. The result demonstrates that* DBN* is more suitable for recognizing data generated by mathematical expression, because* DBN* can automatically extract features from the data, layer by layer, and is similar to composition of formula which is constituted by its subformulas.

**Figure 6:**
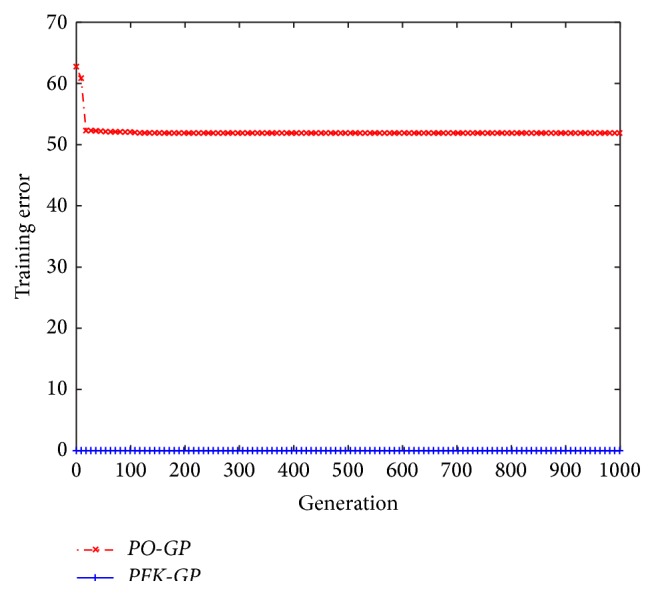
The evolutionary result of *P*_13_ with* PO-GP* and* PFK-GP*.

We set parameters in* GP* with Pareto optimization (*PO-GP*) [29] and* PFK-GP* as shown in Table 3. For data generated by *P*_13_ (see Table 7, coefficient *z* is −2.098, *a* is −2.998),* PO-GP* and* PFK-GP* deal with the SR problem, respectively. The result was illustrated as Figure 6, where

(2) $$\begin{array}{c} \text{Trainerror}=\sqrt{\text{mean}\left({\left(y\text{train}-y\text{predtrain}\right)}^{2}\right)}. \end{array}$$

We could find that after processing test data of formula model *P*_13_,* PFK-GP* found its best model at the first generation and its fitness is higher, while* PO-GP* found its best model until 718th generation and its fitness is much lower than that in* PFK-GP*. The* PFK-GP* can get the right formulas quickly because the model *P*_13_ recognized by the algorithm* GenerateFs* is inserted into the initialized population of evolutionary computation. For the formula models whose characteristics are consistent with data features in* PFKB*, they can be recognized with high probability and can be combined into population of* PFK-GP*. The* PFK-GP* can firstly search the coefficients in these formula models and get the mathematical expression with good fitness value. Therefore, the algorithm* GenerateFs* can speed up the process of* PFK-GP* dealing with SR and can improve the accuracy of SR results.

In order to test whether* PFK-GP* can overcome overfitting or not, a dataset is created by *E*_1_ which has not existed in the training models of* PFKB*. The two algorithms* PO-GP* and* PFK-GP* are, respectively, applied to process the dataset. The two algorithms, which run, respectively, 100 and 1000 generations, have similar convergence curves in Figure 8. However,* PFK-GP* can find better fitness results compared with* PO-GP*, because* PFK-GP* searches fitted solution in the space includes more functions whose data features are in accord with *E*_1_. Since the initial population, which is generated by the algorithms (*CountSamePartF* and* CountSpecU*) in* PFKB*, contains subformulas in formula models which are recognized by* PFKB* and represents data features of these subformulas,* PFK-GP* can find the right formulas which are more fitted to the raw dataset.

**Figure 7:**
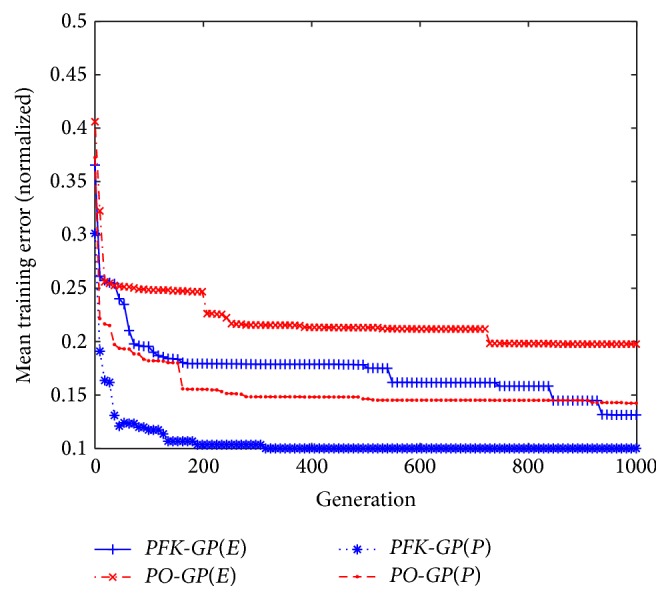
Average results from six groups of means training errors in* PO-GP* and* PFK-GP*.

**Figure 8:**
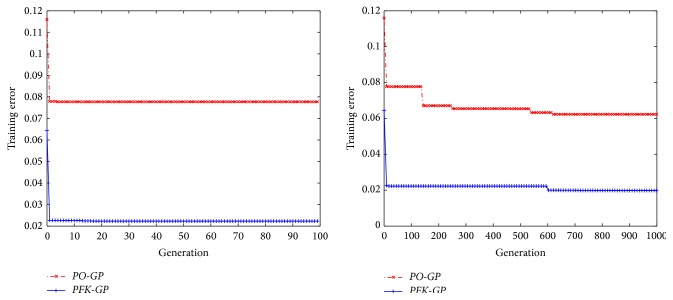
The SR evolutionary process of *E*_1_ with* PO-GP* or* PFK-GP* under different generations.

**Figure 9:**
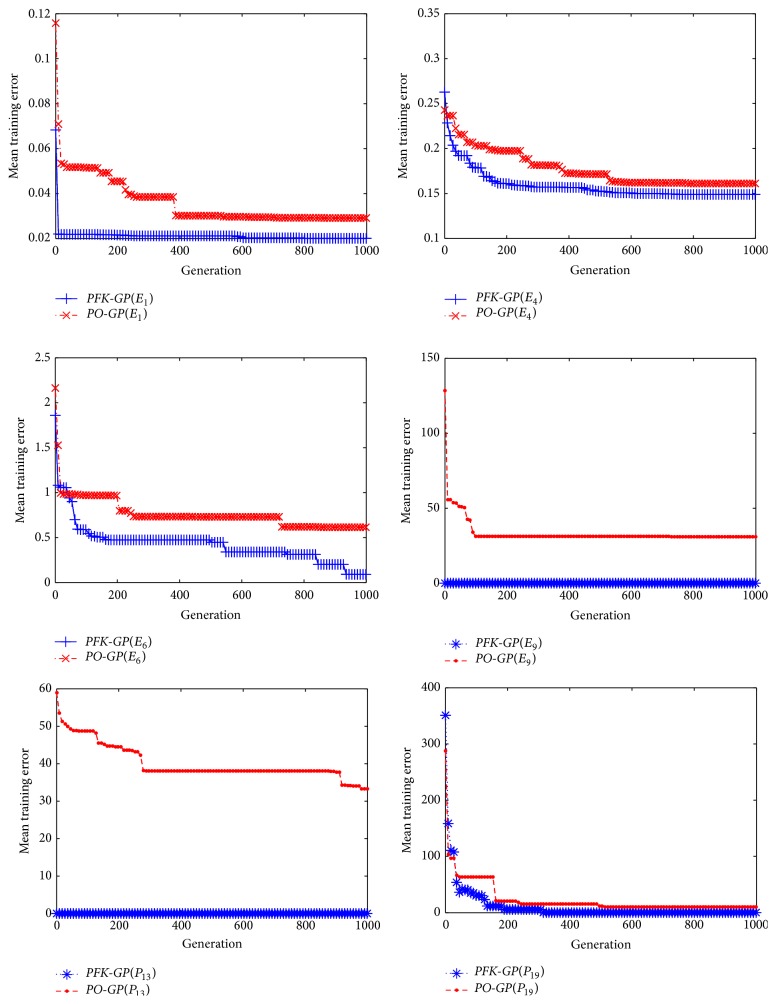
Training error results in which six datasets generated by *E*_1_, *E*_4_, *E*_6_, *P*_1_, *P*_13_, and *P*_19_ dealt with* PO-GP* and* PFK-GP,* respectively.

In order to observe overall performance of the* PFK-GP*, we select six datasets as testing set. Three of them generated by formula models (*P*_9_, *P*_13_, *P*_19_) from Table 7 are involved in the process of training* DBN*, while the other three generated by formula models (*E*_1_, *E*_4_, *E*_6_) from Table 5 are not involved in that process. The two algorithms* PFK-GP* and* PO-GP* are executed, respectively, ten times in order to gain the right formulas from the six different datasets. The six results of mean training error gained by the two algorithms are shown in Figure 9. And the average results from six groups of mean training errors are listed in Figure 7. The* PFK-GP(E)* and* GP*(*E*) are the average results of *E*_1_, *E*_4_, and *E*_6_, while* PFK-GP(P)* and* PO-GP(P)* are the average results of *P*_9_, *P*_13_, and *P*_19_. We can conclude that the comprehensive performance of the* PFK-GP* is better than that of the* PO-GP* based on the results in Figures 7 and 9, because the algorithm* PFK-GP* utilizes the method* GenerateFs* to find the fitted formula model directly and the methods* CountSamePartF* and* CountSpecU* to identify subformula models which have data features consistent with test set. The best mathematical expressions* PFK-GP* and* PO-GP* found are listed in Table 4.

In order to measure relativity between experimental data and predictive data, the formula Training Variation Explained (TVE) is defined as follows:

(3) $$\begin{array}{c} \text{TVE}=1-\frac{\text{sum}\left({\left(y\text{train}-y\text{predtrain}\right)}^{2}\right)}{\text{sum}\left({\left(y\text{train}-\text{mean}\left(y\text{train}\right)\right)}^{2}\right)}. \end{array}$$

**Figure 10:**
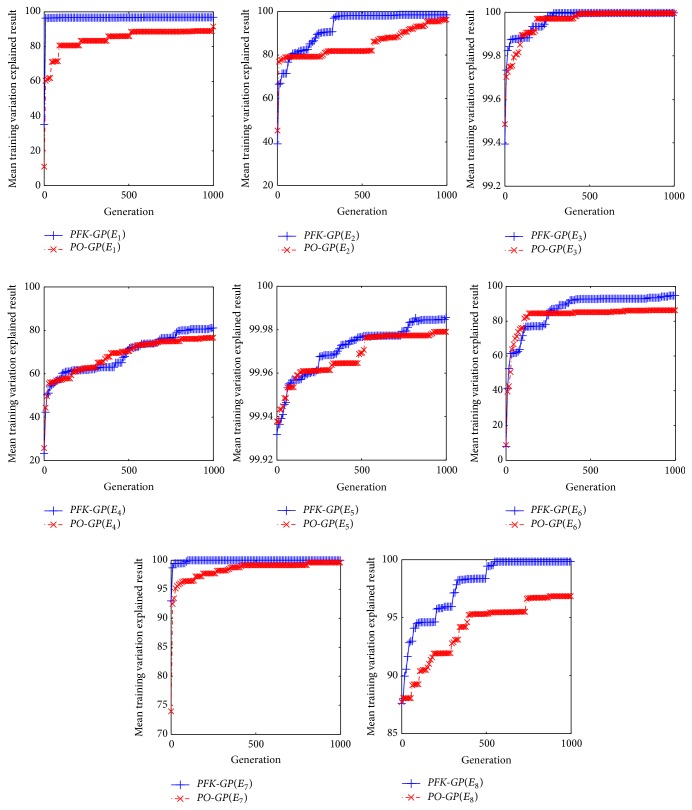
TVE for models *E*_1_–*E*_8_ contrast the random population in traditional* PO-GP* with the* PFK-GP*.

**Figure 11:**
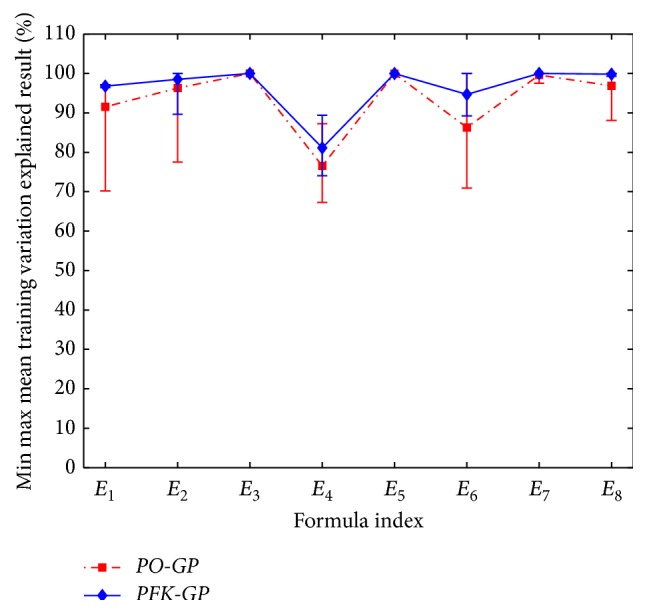
TVE results of* PO-GP* compared with* PFK-GP* in eight formula models.

The higher the TVE value, the more valid the predictive data.* PO-GP* and* PFK-GP* are run ten times, respectively, in the dataset generated from eight prediction models (see Table 6 *E*_1_–*E*_8_). The eight results of different dataset processed by the above two algorithms are listed in Figure 10. And the maximum, minimum, and average results of TVE are listed in Figure 11. From the results in the two figures, the formulas that* PFK-GP* finds are more relative to the experimental formula models than those* PO-GP* finds.

## 5. Related Work

The search space of SR is huge even for rather simple basis functions [31]. In order to avoid search space that is too far from the desired output range determined by the training dataset, the interval arithmetic [34] and the affine arithmetic [26], which can compute the bounds of* GP* tree expression, are imported into SR. Although the method based on affine arithmetic can generate the tighter bounds of the expression in comparison with the interval arithmetic method, its accuracy often leads to high computational complexity [35]. Moreover, the size of search space is still huge because there are plentiful candidate expressions which fit to the data bound computed by the above two arithmetic methods.

In addition to the above arithmetic method, machine learning methods are used to compact or reduce the search space of SR. FFX technology uses pathwise regularized learning algorithm to rapidly prune a huge set of candidate basis functions down to compact model based on the generalization linearly model (GLM); hence the technology outperforms GP-SR in speed and scalability due to its simplicity and deterministic nature [8]. However, it may abandon correct expressions and make them not in the space of GLM. A hybrid deterministic GP-SR algorithm [36] is proposed to overcome the problem of missing correct expression. The hybrid algorithm extracts candidate basis expressions by using FFX and inputs the expressions into the GP-SR. The hybrid algorithm utilizes the candidate expression generated by the linear regression method (pathwise regulation), while our algorithm utilizes the candidate expression by applying the algorithms* CountSamePartF, GenerateFs,* and* CountSpecU*.

By applying expectation-maximization (EM) framework to SR, the clustered SR (CSR) can identify and infer symbolic repression of piecewise function from unlabelled, time-series data [9]. The CSR can reduce the space of searching piecewise function owing to the fact that the EM can search simultaneously the subfunction parameters and latent variables that represent the information of function segment. The abstract expression grammar (AEG) SR is proposed to perform the process of genetic algorithm (GA), allowing user control of the search space and the final output formulas [37]. On understanding the given application, users can specify the goal expression of SR and limit the size of search space by using abstract expression grammars. Compared with manually assigning expression and limiting the search space with AEGSR, in the paper, the methods about PFK can automatically extract the candidate expression from dataset by using statistical method and dynamically adjust the search space by using* GP*.

The methods that inject prior or expert knowledge in evolutionary search [12, 13] are introduced to find effective solutions that can show mathematical expression more compactable and interpretable. In these papers, the prior and expert knowledge are the solutions which are mathematical expressions in some applications. The knowledge is merged into* GP* by inserting randomized pieces of the approximate solution into population. One of the major differences between these methods and our method is how prior or expert knowledge is created. The knowledge in [12, 13] is the existing formula model that comes from the previous solutions and can be called static knowledge. However, the knowledge in our method is the formula model which is consistent with data features that are originated from the algorithms* GenerateFs, CountSamePartF,* and* CountSpecU* and can be called dynamical knowledge that is changed with the features of test dataset. Therefore, our methods can insert more suitable knowledge into the* GP*.

## 6. Conclusion

In this paper, a* PFK-GP* method is proposed to deal with the problem of symbolic regression based on analyzing the process of how a researcher constructs a mathematical model. The method can understand experimental data features and can extract some formulas consistent with experimental data features. In order to implement the function of understanding data features,* PFK-GP*, through the* DBN* method, firstly creates* PFKB* that can extract features from test dataset generated by training formula models. The experiment results confirm, compared with SVM, that* DBN* can produce better results that extract features from formula models and classify test data into its corresponding formula model. Then, the methods of classification and recognition are implemented to find some formula models that are similar or related to experimental data features as much as possible. For the classification, we exploit the algorithm* GenerateFs* based on* DBN* to match the experimental data with formula models in* PFKB*. With regard to recognition, we propose the algorithms of* CountSamePartF* and* CountSpecU* to obtain some subformula models which have local features consistent with experimental data. The classification can help* PFKB* to find formula models that are consistent with whole data features while the recognition can help* PFKB* to find subformula models consistent with local data features. At last, the algorithm* randomGenP* is used to generate individuals of evolutionary population according to the result of the above three algorithms. Through combining and transforming these individuals,* GP* can automatically obtain approximate formulas that are best fitting to the experimental data.

Compared with Pareto* GP*,* PFK-GP*, which is built on the* PFKB* with the functions of classification and recognition, can explore formulas in the search space of data features. So, it can accelerate the speed of convergence and improve the accuracy of formula obtained.

Obviously, the high efficiency of* PFK-GP* depends on the powerful methods of classification and recognition based on* PFKB*. Therefore, it is an important part of the future work to improve the accuracy of the above two methods. The two methods depend on the representation of data features of formula model. In the paper, the two assumptions based on statistics and counts are used to obtain the formulas which can show the data features. The features of formula model are not defined explicitly. And the two assumption are not proved by formal proofs. There are some uncertainties in those assumptions. Therefore, the new representation which can show whole or local features of formula models will be researched to find formulas which can better fit to experiment data. In addition, the rules of formulas transforming and inferring that are similar to researchers' methods will be explored in the evolution of* GP*.

## Figures and Tables

**Algorithm 1:**
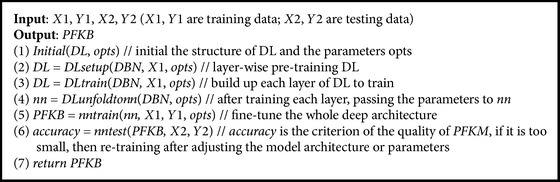
*TrainDBN*: training *DBN* to generate the *PFKB*.

**Algorithm 2:**
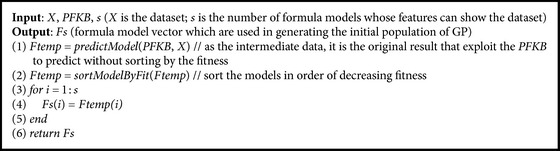
*GenerateFs*.

**Algorithm 3:**
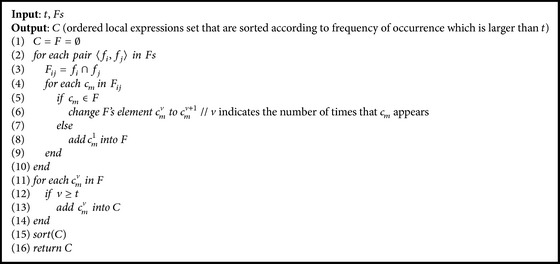
*CountSamePartF*.

**Algorithm 4:**
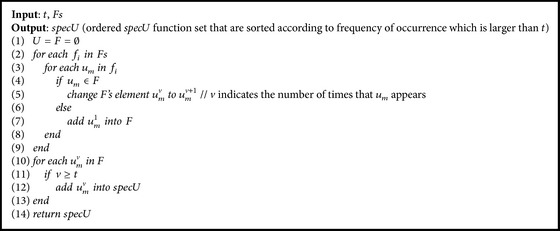
*CountSpecU*.

**Algorithm 5:**
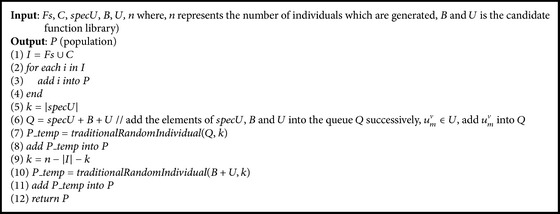
*randomGenP*.

**Algorithm 6:**
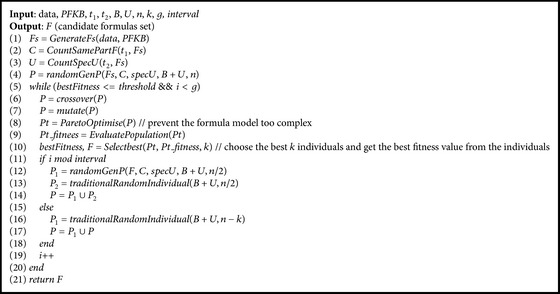
*PFK-GP*.

**Table 1:** The result of *U* in *Fs* computed by algorithm *CountSpecU*  (see Algorithm 4).

*u* in *Fs*	Frequency of occurrence
tan	3
cos	2
sqrt	1
exp	1
log	1

**Table 2:** The parameter values in algorithm of *DBN* and SVM.

*DBN* parameters	Value	SVM parameters	Value
The number of *DBN* layers	4	svm_type	c-svc
The size of *DBN* hide nodes	50	Kernel	Gaussian
The number of epochs	200	Gamma	0.07
Batch size	40	Coef	0
Momentum	0	Cost	1.0
Alpha	1	Degree	3.0
activation_function	sigm	Shrinking	1

**Table 3:** Parameter values in *GP* and *PFK*-*GP*.

Parameter	Value
	GPTIPS [29] multigene syntax
Representation	Number of genes: 1
	Maximum tree depth: 5
Population size	50
Number of generations	1000
Selection	Lexicographic tournament selection
Tournament size	3
Crossover operator	Subtree crossover
Crosser probability	0.85
Mutation operator	Subtree mutation
Mutation probability	0.1
Reproduction probability	0.05
Fitness	$\frac{1}{N}\sqrt{\sum {\left(y-\hat{y}\right)}^{2}}$
Elitism	Keep 1 best individual

**Table 4:** The best mathematical expression of *PFK-GP* finding.

Number	The best mathematical expression
*E*_1_	*y* = 0.7001*∗*tan⁡(*x*_1_ *∗x*_4_ − 5.049) − 0.7001 *∗* *x*_1_ *∗*cube⁡(2.575)*∗*cube⁡(*x*_4_) − 1.001
*E*_2_	*y* = 7.214*∗*sin⁡(*x*_1_) + 1.001*∗*tan⁡(*x*_2_)
*E*_3_	*y* = 0.25*∗*square⁡(*x*_1_ + *x*_2_ − 6) − 0.2179
*E*_4_	$y=1.001-\frac{\left(1.332*\left(4*x_{1}+\log\left(\mathrm{square}\left(x_{2}\right)\right)\right)\right)}{\left(2*\mathrm{square}\left(x_{2}\right)+5.585\right)}$
*E*_5_	*y* = *x*_2_ − 2.092tanh⁡(square⁡(sin⁡(*x*_1_))) + 0.8795
*E*_6_	*y* = 6*∗*cos⁡(*x*_2_)*∗*sin⁡(*x*_1_) − 0.00444
*E*_7_	*y* = sin⁡(*x*_1_) − 6*∗x*_1_ + square⁡(*x*_1_) + 14
*E*_8_	*y* = log⁡(*x*_2_) + sqrt⁡(*x*_1_) + sin⁡(*x*_1_) + 0.1823

**Table 5:** Test data used in *PFK-GP*.

Number	Formula
*E*_0_	$y=-1.97+1.25*\frac{\text{sqrt}\left(x_{0}\right)}{\tan\left(x_{1}\right)}+\frac{\tan\left(x_{0}\right)}{\exp\left(x_{1}\right)}+\cos\left(x_{0}\right)$ *∗*sin⁡(*x*_1_)
*E*_1_	*y* = exp⁡(2*∗x*_1_ *∗*sin⁡(*pi∗x*_4_)) + sin⁡(*x*_2_ *∗x*_3_)
*E*_2_	*y* = 3.56 + 7.23*∗*sin⁡(*x*_0_) + tan⁡(*x*_3_)
*E*_3_	*y* = (*x*_0_ − 3)*∗*(*x*_3_ − 3) + 2*∗*sin⁡((*x*_0_ − 4)*∗*(*x*_3_ − 4))
*E*_4_	$y=\frac{\left(\mathrm{quart}\left(x_{1}-3\right)+\mathrm{cube}\left(x_{2}-3\right)-\left(x_{2}-3\right)\right)}{\left(\mathrm{quart}\left(x_{2}-4\right)+10\right)}$
*E*_5_	*y* = tanh⁡(cos⁡(2*∗x*_0_)) + *x*_3_
*E*_6_	*y* = 6*∗*sin⁡(*x*_0_)*∗*cos⁡(*x*_3_)
*E*_7_	*y* = sin⁡(*x*_0_) + square⁡(*x*_0_) + 5
*E*_8_	*y* = sqrt⁡(*x*_0_) + log⁡(1.2*∗x*_3_) + sin⁡(*x*_0_)

**Table 6:** Test data of two algorithms *CountSamePartF* and *CountSpecU*  (see Algorithms 3 and 4).

Number	Formula
*T*_1_	*P*_1_
*T*_2_	*P*_2_
*T*_3_	*P*_3_
*T*_4_	*P*_4_
*T*_5_	*P*_5_
*T*_6_	$y=z+a*\frac{\mathrm{sqrt}\left(x_{0}\right)}{\tan\left(x_{1}\right)}+\sin\left(x_{1}\right)$
*T*_7_	$y=z+a*\frac{\mathrm{sqrt}\left(x_{0}\right)}{\tan\left(x_{1}\right)}+x_{1}$
*T*_8_	$y=z+a*\frac{\tan\left(x_{0}\right)}{\exp\left(x_{1}\right)}+\cos\left(x_{0}\right)*\sin\left(x_{1}\right)+\sin\left(x_{1}\right)$
*T*_9_	$y=z+a*\frac{\tan\left(x_{0}\right)}{\exp\left(x_{1}\right)}+\cos\left(x_{0}\right)*\sin\left(x_{1}\right)+x_{1}$
*T*_10_	*y* = *z* + *a∗*cos⁡(*x*_0_)*∗*sin⁡(*x*_1_) + square⁡(*x*_1_)

**Table 7:** Training data of algorithm *TrainDBN*  (see Algorithm 1).

Number	Formula
*P*_1_	*y* = *z* + *a∗x*_0_
*P*_2_	$y=z+\left(a_{1}*\frac{\left(x_{3}+x_{1}\right)}{\left(a_{2}*x_{4}\right)}\right)$
*P*_3_	$y=z+\left(a_{1}*\left(\frac{\left(\left(x_{3}-x_{0}\right)+\left(x_{1}/x_{4}\right)\right)}{\left(a_{2}*x_{4}\right)}\right)\right)$
*P*_4_	*y* = *z* + *a∗*sin⁡(*x*)
*P*_5_	*y* = *z* + *a∗*log⁡(*x*)
*P*_6_	*y* = *z* + *a∗*sqrt⁡(*x*)
*P*_7_	*y* = *z* − (*a*_1_ *∗*exp⁡(*a*_2_ *∗x*_0_))
*P*_8_	*y* = *z* + (*a*_1_ *∗*sqrt⁡(*a*_2_ *∗x*_0_ *∗x*_3_ *∗x*_4_))
*P*_9_	$y=\left(\left(\frac{\left(a_{1}*\mathrm{sqrt}\left(x_{0}\right)\right)}{\left(a_{2}*\log\left(x_{1}\right)\right)}\right)*\left(\frac{\left(a_{3}*\exp\left(x_{2}\right)\right)}{\left(a_{4}*\mathrm{square}\left(x_{3}\right)\right)}\right)\right)$
*P*_10_	$y=z+\left(a_{1}*\left(\frac{\left(\left(a_{2}*x_{1}\right)+\left(a_{3}*\mathrm{square}\left(x_{2}\right)\right)\right)}{\left(a_{4}*\mathrm{cube}\left(x_{3}\right)\right)+\left(a_{5}*\mathrm{quart}\left(x_{4}\right)\right)}\right)\right)$
*P*_11_	*y* = *z* + (*a*_1_ *∗*cos⁡(*a*_2_ *∗x*_0_ *∗x*_0_ *∗x*_0_))
*P*_12_	*y* = *z* − (*a*_1_ *∗*(cos⁡(*a*_2_ *∗x*_0_)*∗*sin⁡(*a*_3_ *∗x*_4_)))
*P*_13_	$y=z-\left(a*\left(\left(\frac{\tan\left(x_{0}\right)}{\tan\left(x_{1}\right)}\right)*\left(\frac{\tan\left(x_{2}\right)}{\tan\left(x_{3}\right)}\right)\right)\right)$
*P*_14_	$y=z-\left(a*\left(\left(\cos\left(x_{0}\right)-\tan\left(x_{1}\right)\right)*\left(\frac{\tanh\left(x_{2}\right)}{\sin\left(x_{3}\right)}\right)\right)\right)$
*P*_15_	$y=z-\left(a*\left(\left(\frac{\tan\left(x_{0}\right)}{\exp\left(x_{1}\right)}\right)*\left(\log\left(x_{2}\right)-\tan\left(x_{3}\right)\right)\right)\right)$
*P*_16_	*y* = *a∗x*_3_
*P*_17_	*y* = *a*_1_ *∗x*_1_ + *a*_2_ *∗x*_4_
*P*_18_	$y=\frac{\mathrm{sqrt}\left(x_{2}\right)}{\tan\left(x_{5}/a\right)}$
*P*_19_	$y=\frac{\cos\left(x_{2}\right)}{\mathrm{cube}\left(x_{5}/a\right)}$
*P*_20_	*y* = tanh⁡(*x*_2_ *∗a∗*cube⁡(*x*_5_ + abs⁡(*x*_1_)))
*P*_21_	*y* = tanh⁡(abs⁡(*x*_2_ *∗a* + *x*_5_)*∗*cube⁡(*x*_5_ + abs⁡(*x*_1_)))
*P*_22_	$y=\tanh\left(\tan\left(\frac{x_{5}}{a}\right)*\mathrm{cube}\left(x_{5}+\mathrm{abs}\left(x_{1}\right)\right)\right)$
*P*_23_	*y* = tanh⁡(cos⁡(*x*_2_ *∗a*)*∗*cube⁡(sqrt⁡(*x*_2_)))
*P*_24_	*y* = tanh⁡(cos⁡(*x*_2_ *∗a*)*∗*cube⁡(*x*_5_ + abe⁡(*x*_1_)))
*P*_25_	*y* = *z*
*P*_26_	*y* = *z* + *x*_2_
*P*_27_	y=z+x2x0∗x2z+x2x0∗x2XXX
*P*_28_	$y=\left(\frac{\left(x_{0}-z_{1}\right)}{\left(x_{0}+x_{2}\right)}\right)*\left(\frac{\left(x_{5}-z_{2}\right)}{\left(x_{0}*a_{1}\right)}\right)$
*P*_29_	*y* = *a∗*sqrt⁡(*x*)
*P*_30_	*y* = *a∗*log⁡(*x*)
*P*_31_	*y* = *a∗*square⁡(*x*)
*P*_32_	*y* = *a∗*tanh⁡(*x*)
*P*_33_	*y* = *a∗*sin⁡(*x*)
*P*_34_	*y* = *a∗*cos⁡(*x*)
*P*_35_	*y* = *a∗*exp⁡(*x*)
*P*_36_	*y* = *a∗*cube⁡(*x*)
*P*_37_	*y* = *a∗*quart⁡(*x*)
*P*_38_	*y* = *a∗*tan⁡(*x*)
*P*_39_	*y* = *a∗*abs⁡(*x*)
